# The type-material of Arctiinae (Lepidoptera, Erebidae) described by Burmeister and Berg in the collection of the Museo Argentino de Ciencias Naturales Bernardino Rivadavia (Buenos Aires, Argentina)

**DOI:** 10.3897/zookeys.421.6666

**Published:** 2014-06-27

**Authors:** Hernán M. Beccacece, Benoit Vincent, Fernando R. Navarro

**Affiliations:** 1 Centro de Investigaciones Entomológicas de Córdoba, Instituto de Investigaciones Biológicas y Tecnológicas - CONICET, Av. Vélez Sársfield 1611, 5016, Córdoba, Argentina; 2 1 rue Roger Rameau, F – 93110 Rosny sous Bois. Correspondant au Muséum national d’Histoire naturelle, département Systématique et Evolution, CP 50 (Entomologie), F – 75231 Paris cedex 05; 3 Instituto Superior de Entomología “Dr. Abraham Willink” (UNT) - CONICET, Miguel Lillo 205, 4000, San Miguel de Tucumán, Tucumán, Argentina

**Keywords:** MACN, type specimens, Lepidoptera, Erebidae, Arctiinae, Neotropics

## Abstract

Carlos G. Burmeister and Carlos Berg were among the most important and influential naturalists and zoologists in Argentina and South America and described 241 species and 34 genera of Lepidoptera. The Museo Argentino de Ciencias Naturales Bernardino Rivadavia (MACN) housed some of the Lepidoptera type specimens of these authors. In this study we present a catalogue with complete information and photographs of 11 Burmeister type specimens and 10 Berg type specimens of Phaegopterina, Arctiina and Pericopina (Lepidoptera, Erebidae, Arctiinae, Arctiini) housed in the MACN. Lectotypes or holotypes were designated where primary type specimens could be recognized; in some cases we were not able to recognize types. The catalogue also proposes nomenclatural changes and new synonymies: *Opharus picturata* (Burmeister, 1878), **comb. n.**; *Opharus brunnea* Gaede, 1923: 7, **syn. n.**; *Hypocrisias jonesi* (Schaus, 1894), **syn. n.**; *Leucanopsis infucata* (Berg, 1882), **stat. rev.**; *Paracles argentina* (Berg, 1877), **sp. rev.**; *Paracles uruguayensis* (Berg, 1886), **sp. rev.**

## Introduction

**Figure 1.**
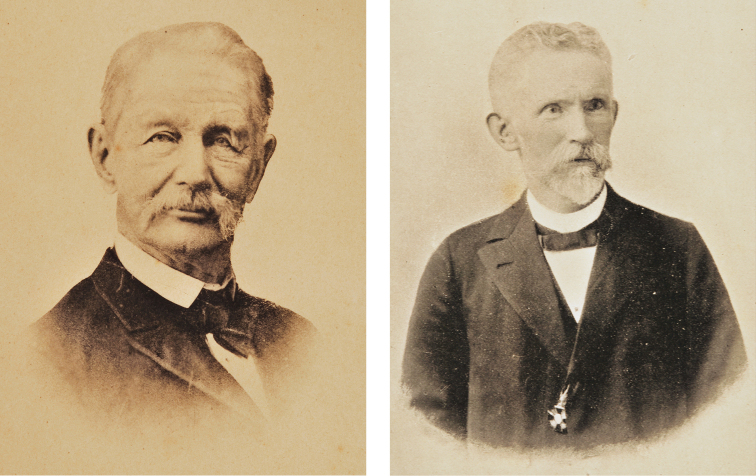
Portraits of Carlos Burmeister (approximately 1885) (Left) and Carlos Berg (undated) (Right) taken from Berg (1895b) and Gallardo (1902), respectively.

Karl Hermann Konrad Burmeister or Carlos Germán Conrado Burmeister (15.I.1807–02.V.1892) and Friedrich Wilhelm Karl Berg or Carlos Berg (02.IV.1843–19.I.1902) (Fig. 1) were among the most important and influential zoologists in Argentina and were directors of the Museo Argentino de Ciencias Naturales Bernardino Rivadavia (MACN).

Burmeister was born in Stralsund (Prussia, current-day Germany) and followed his studies there until 1825. He obtained his doctorate in medicine in 1829 at the University of Halle (Germany). He taught as a professor of zoology beginning in 1837. Thanks to the support of his protector Alexander von Humboldt, he made a voyage of exploration in Brazil from September 1850 to March 1852. Then he had a mission in Argentina and Uruguay from 1856 to 1860. He resigned his professorship in zoology in 1861 to become director of the MACN. With just a museum of curiosities, he contributed to make it a significant scientific institution. A fall from a ladder and broken glass of a showcase exhibit in the museum caused him serious injuries in February 8, 1892. He recommended Carlos Berg to the Government of the Republic of Argentina as his successor as Director of the MACN. Carlos Burmeister died due to his injuries May 2, 1892. Among his many publications (about 270 articles), he published his manual of entomology in 5 volumes (1832–1855) and “*Description physique de la République Argentine: d’après des observations personnelles et étrangères*” in 4 volumes (1876–1886). For the order Lepidoptera, between 1854 and 1880, he described and named over 13 genera and 119 species.

Around 1872, Professor Burmeister searched for entomologists to fill the newly created position of inspector at the MACN. He contacted in particularly his German colleagues, who recommended Carlos Berg. Berg was born in Tukums (Russia, current-day Latvia). Fascinated by natural history since his childhood, he started in the trade and worked as a librarian. In 1870, he became curator of the museum of Riga (Latvia). For medical reasons, he had to leave Riga and reside in warmer climates. Around 1872, he assumed his post at MACN in June 1873 and became professor of Natural History at the National College of Buenos Aires in March 1876. In 1890, he was responsible for organizing the National Museum of Montevideo, Uruguay. In April 1892, on the recommendations of Burmeister, who was side-lined by his injury, Berg was named Director of the MACN. During the 10 years of his leadership, he reorganized all collections by making many adjustments in identifications and labelling. Much of the material studied by Berg came from his expeditions to Patagonia (1874), Cordoba and Catamarca (1875), Corrientes and Misiones (1876–1877), San Luis, Mendoza and Chile (1878–1879) as well as many trips around Buenos Aires and Uruguay. He published on entomology, other zoological groups, paleontology and botany (about 200 articles). One of the most important Lepidoptera publication was “Lepidopteros patagónicos observados en el viaje de 1874” (1875), also translated into German simultaneously in the Bulletin de la Societe Imperiale des Naturalistes de Moscou. Berg described and named over 21 genera and 122 species of Lepidoptera between 1875 and 1901.

Further detail about Burmeister’s and Berg’s lives can be obtained respectively in Berg (1895a, b) and Gallardo (1902).

## Methods and materials

The types of the nominal taxa described by Burmeister and Berg and preserved in MACN have been recognized as types with the exception of some taxa that could not be identified. A number of Arctiina, Phaegopterina and Pericopina types described by Burmeister and Berg, presumed to be in MACN, are still missing and are probably lost.

**Figure 2.**
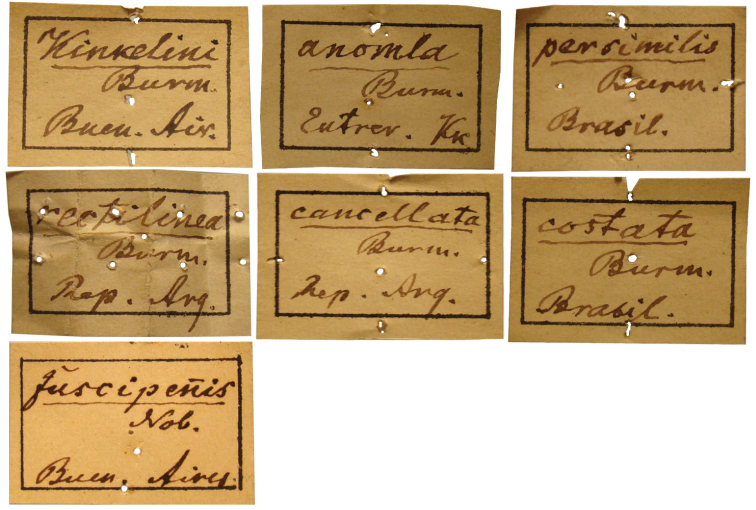
Square labels handwritten by Burmeister. These are the main labels preceding the specimen series.

**Burmeister types.** Each taxon represented in the Burmeister collection carries a square white label with a black frame on which the genus and/or species is underlined. Some labels have his last name abbreviated as “Burm.” or in full “Burmeister,” the country abbreviated as “R. A.” or “Rep. Arg.” [Republic of Argentina] and sometimes the city or town is stated and the initials “Nob.” [Nobis] (Fig. 2). These labels were pinned to the bottom of the box with one or two short pins at its upper and lower edge, or at the middle. The specimens were placed below this label. In some cases, the white square label was pinned to the first specimen.

Labels. A great disadvantage of the Burmeister collection is that the specimens are not labelled as types, so we had to find additional evidence to associate these specimens with the original type series.

Arrangement. The type material of Burmeister’s species are placed as follows: six species in the Burmeister collection, four species in the Berg collection and one species in both collections.

**Figure 3.**
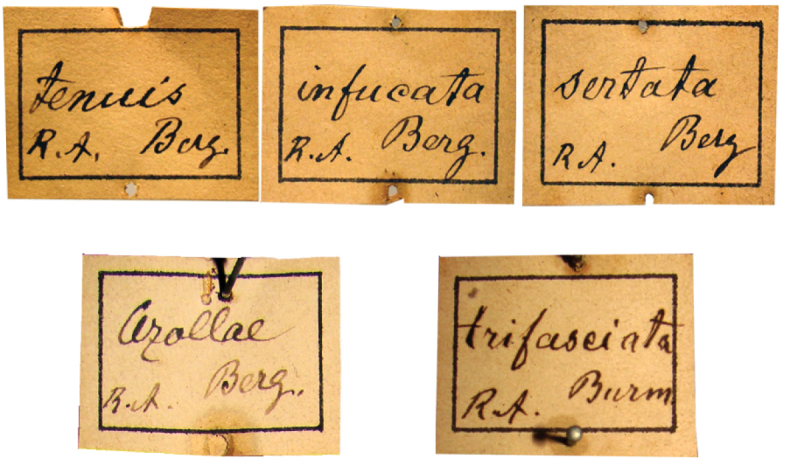
Square labels handwritten by Berg. These are the main labels preceding the specimen series.

**Figure 4.**
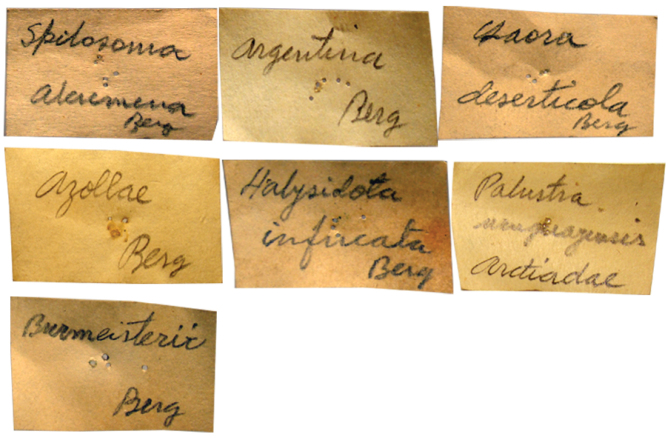
Labels handwritten by Berg in pencil. These labels are pinned on type specimens.

**Berg types.** Each taxon represented in the Berg collection carries a square white label with a black frame with the genus and another with the same form with the species (Figure 3). These labels, like Burmeister’s, were pinned to the bottom of the box with one pin at the middle of the upper and lower edge. The specimens were placed below these labels. A white label with “Berg” handwritten in pencil with the genus, the species’ name, or the binominal name, is pinned to the type specimen (Figure 4).

Labels. The type specimen has a label that identifies them as such; only two species are unlabelled.

Arrangement. The type material of Berg’s species are placed as follows: six species in the Berg collection, one in the Burmeister collection and three species in both collections.

Burmeister’s entomological collection was held in Martin-Luther-Universitat, Wissenschaftsnbereich Zoologie, Halle (MLUH) until he resigned his position as professor of zoology and travelled to Argentina in 1861. Since then, his collection has been kept in MACN. Burmeister described neotropical Arctiinae since 1878. Therefore, all Burmeister type specimens should be in MACN as well as those described by Berg. Berg’s entomological collection is placed entirely in this institution.

When no indication of the number of specimens examined was given by Berg or Burmeister in the original description, we treated the specimens as syntypes even if only a single specimen was found. For nomenclature stability, and in order to establish the identity of several species-group names, we assign lectotypes.

### Acronyms

MACN Museo Argentino de Ciencias Naturales Bernardino Rivadavia, Buenos Aires, Argentina.

ZMHB Zoologisches Museum, Humboldt Universität, Berlin, Germany.

USNM National Museum of Natural History, formerly United States National Museum, Washington DC, United States of America.

## Results

**Figure 5.**
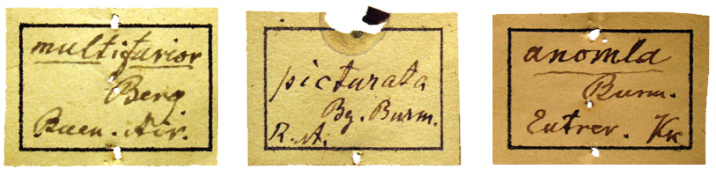
Labels handwritten by Burmeister with other authors’ names. These are the main labels preceding the specimen series.

There are some square labels handwritten by Burmeister that include the last name of Karl Berg as, for instance, the description of *Antarctia multifarior* (Burmeister, 1878) (Fig. 5). Moreover, on the square label handwritten by Burmeister of *Halesidota* (sic) *picturata* (Burmeister, 1878) the abbreviation of both authors’ last names appears as “Bg.” [Berg] and “Burm.” [Burmeister]. In the original description, the genus and specific names are followed by Berg (as in the author’s description) but there are no other previous publications, so the specific name is assigned by Burmeister. The same problem occurs with *Ecpantheria anomala* (Burmeister, 1883); on the label handwritten by Burmeister, the abbreviation “Kk.” [Kinkelin] appears as well as the original description (Fig. 5). Possibly, he placed the name in honor of the collector. In his publication, Burmeister indicated the author and year of the original description below the genus and specific name only if he included a redescription.

**Catalogue of type specimens.** All species of the subtribes Phaegopterina, Arctiina and Pericopina (Arctiinae) described by Burmeister and Berg found in MACN are listed below in the systematic order of the catalogue of the neotropical Arctiinae (Watson and Goodger 1986). For each taxon, the following entries are given:

Original combination. The taxon appears in the text of the original description.

Current identity. The currently accepted classification of the taxon before this article.

Material. The status of the specimen type (syntype or holotype), the sex, and the number of type specimen as stated in the original description. The potential interpretations of the original description are in brackets as well as additional information.

Type locality. Since there were syntypes from more than one locality, it is important to state clearly what the lectotype locality is, based on information in the original description and on the type labels.

MACN. The number of type specimens, sex (if recognizable), type status and condition of the specimens found in MACN; the text of the labels is in italics and between quotation marks. The interpretations of the text of some labels are between brackets; additional information is between parentheses.

Remarks. Discussions on specimen type or other specimens and on the proposed recombination are provided.

### Arctiinae, Arctiini, Phaegopterina

#### Halesidota picturata

Taxon classificationAnimaliaLepidopteraErebidae

Burmeister, 1878: 442

Fig. 6

##### Current identity.

*Phaegoptera picturata* (Burmeister, 1878).

##### Material.

Described based on two female syntypes. A female from Las Conchas (Buenos Aires, Argentina) collected by M. Ruscheweyh; a female reared by Berg from a caterpillar found in “Bande Orientale de l’Uruguay” [Republic of Uruguay].

##### Type locality.

“Bande Orientale de l’Uruguay” [Republic of Uruguay].

##### MACN.

**Figure 6–9.**
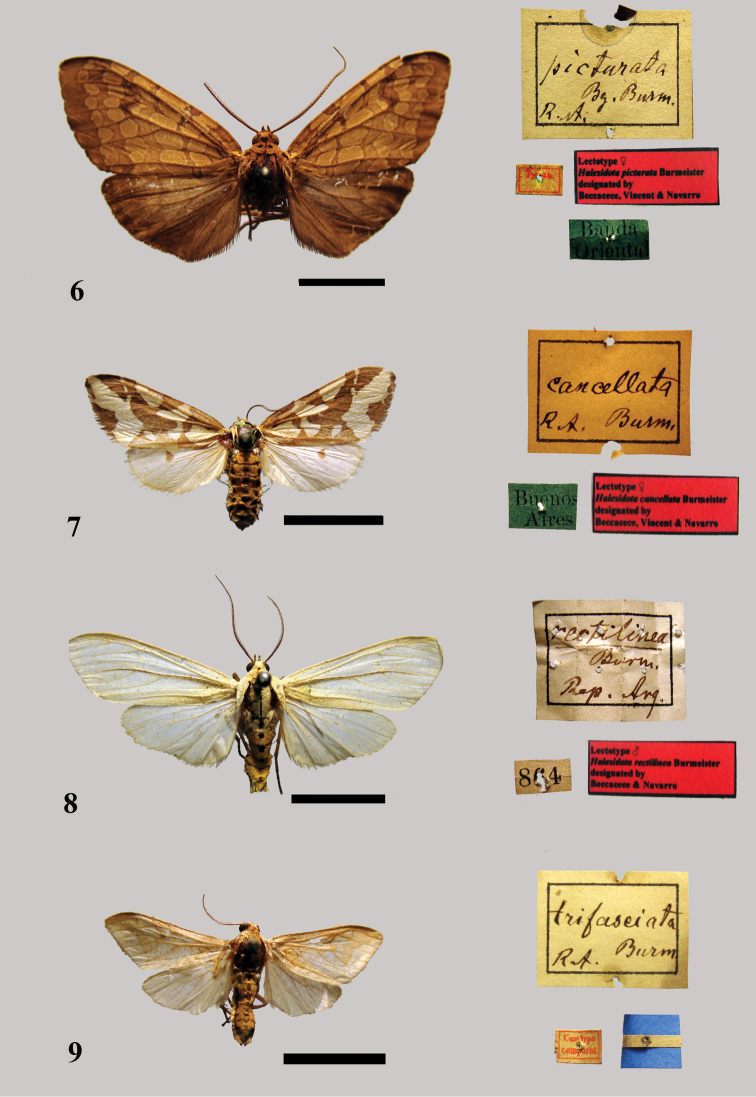
**6** Lectotype female *Halesidota picturata* Burmeister **7** Lectotype female *Halesidota cancellata* Burmeister **8** Lectotype male *Halesidota rectilinea* Burmeister **9** Possible holotype specimen of *Halesidota trifasciata* Burmeister.

A female syntype housed in the Berg collection with a white label with the inscription “Typus,” a green label with the inscription “Banda Oriental” and another red label with the inscription “Lectotype ♀ *Halesidota picturata* Burmeister assigned by Beccacece, Vincent & Navarro.” We hereby designate it as lectotype [MACN]. It is in moderate to poor condition; the abdomen and distal half of the left antenna are missing (Fig. 6).

##### Remarks.

The other female specimen from Las Conchas was not found in the MACN. A male specimen labelled “Cumtypo/comparat.” and “Banda Oriental” is associated with the female lectotype. It possibly corresponds to the same taxon, but it was incorrectly labelled cotype as it was not mentioned in the original description. The female lectotype presents the same habitus as *Opharus brunnea* Gaede, 1923. This taxon was described from a female holotype from the State of Rio Grande do Sul (Brazil), housed in the ZMHB. The proximity of type localities, associated with a similar habitus, particularly in the ornamentation of the forewings, justifies the synonymy of the two taxa. However, the placement of the taxon *picturata* in the genus *Opharus* Walker, 1855 seems more consistent than its current placement in the genus *Phaegoptera* Herrich-Schäffer, [1853]. We therefore propose the following new combination and synonymy.

*Opharus picturata* (Burmeister, 1878), **comb. n.** = *Opharus brunnea* Gaede, 1923: 7, **syn. n.**

#### Halesidota cancellata

Taxon classificationAnimaliaLepidopteraErebidae

Burmeister, 1878: 445

Fig. 7

##### Current identity.

*Tessellota cancellata* (Burmeister, 1878).

##### Material.

Described from an unspecified number of syntypes from Buénos-Ayres [Buenos Aires].

##### Type locality.

Buénos-Ayres [Buenos Aires].

##### MACN.

A female syntype housed in the Berg collection with a green label with the inscription “Buenos Aires” and a red label with the inscription “Lectotype ♀ *Halesidota cancellata* Burmeister designated by Beccacece, Vincent & Navarro.” We hereby designate it as lectotype [MACN]. It is in quite good overall condition, although it is missing the left antenna (Fig. 7). There are two paralectotypes: a male: “Buenos Arres” [Buenos Aires], which is in moderate to good condition; a male: “Buenos Aires” in moderate to poor condition with no antennae, the apices of both forewings are missing and the right hindwing is ripped.

##### Remarks.

There is no distinctive label indicating that these specimens are syntypes. However, the origin of these specimens located in Buenos Aires and their inclusion in the Berg collection, below a square label with Burmeister’s handwriting, leads us to believe that these specimens are syntypes that were studied by Burmeister. Indeed, it seems that Berg and Burmeister frequently exchanged specimens (Roig, pers. comm.). In addition, wing spans indicated in the original description (1–1.2 inches) correspond to the specimens that we consider syntypes.

#### Halesidota rectilinea

Taxon classificationAnimaliaLepidopteraErebidae

Burmeister, 1878: 445

Fig. 8

##### Current identity.

*Biturix rectilinea* (Burmeister, 1878).

##### Material.

Described from an unspecified number of specimens from the interior of the Republic [of Argentina].

##### Type locality.

Interior of the Republic [of Argentina].

##### MACN.

A male syntype included in the Burmeister collection with a square label “*rectilinea* Burm[eister] Rep[ublic] Arg[entina]” (handwritten by Burmeister), a white label with the inscription “864” and a red label with the inscription “Lectotype ♂ *Halesidota rectilinea* designated by Beccacece & Navarro” (Beccacece & Navarro, in press) [MACN]. It is in quite good conditions (Fig. 8).

##### Remarks.

The label with “864” printed on it is the catalogue number in the database of the MACN.

#### Halesidota trifasciata

Taxon classificationAnimaliaLepidopteraErebidae

Burmeister, 1878: 446

Fig. 9

##### Current combination.

*Tessellota trifasciata trifasciata* (Burmeister, 1878).

##### Material.

Described from a single male from Buénos-Ayres [Buenos Aires].

##### Type locality.

Buénos-Ayres [Buenos Aires].

##### MACN.

In the Berg collection there is a male specimen with a label “Cumtypo/comparat.” and another label representing the flag of Argentina without any indication. It could be the holotype specimen; it is in moderate to good conditions with the right antenna missing (Fig. 9).

##### Remarks.

The specimen is associated with a label “*trifasciata* Burm[eister] R[epublic] A[rgentina]” (handwritten by Burmeister).

#### Halesidota fuscipennis

Taxon classificationAnimaliaLepidopteraErebidae

Burmeister, 1878: 441

Fig. 10

Hypocrisias jonesi (Schaus, 1894): 230 (*Phaegoptera*) **syn. n.**

##### Current identity.

*Hypocrisias fuscipennis* (Burmeister, 1878).

##### Material.

Described from an unspecified number of specimens from Buénos-Ayres [Buenos Aires].

##### Type locality.

Buénos-Ayres [Buenos Aires].

##### MACN.

**Figure 10–13.**
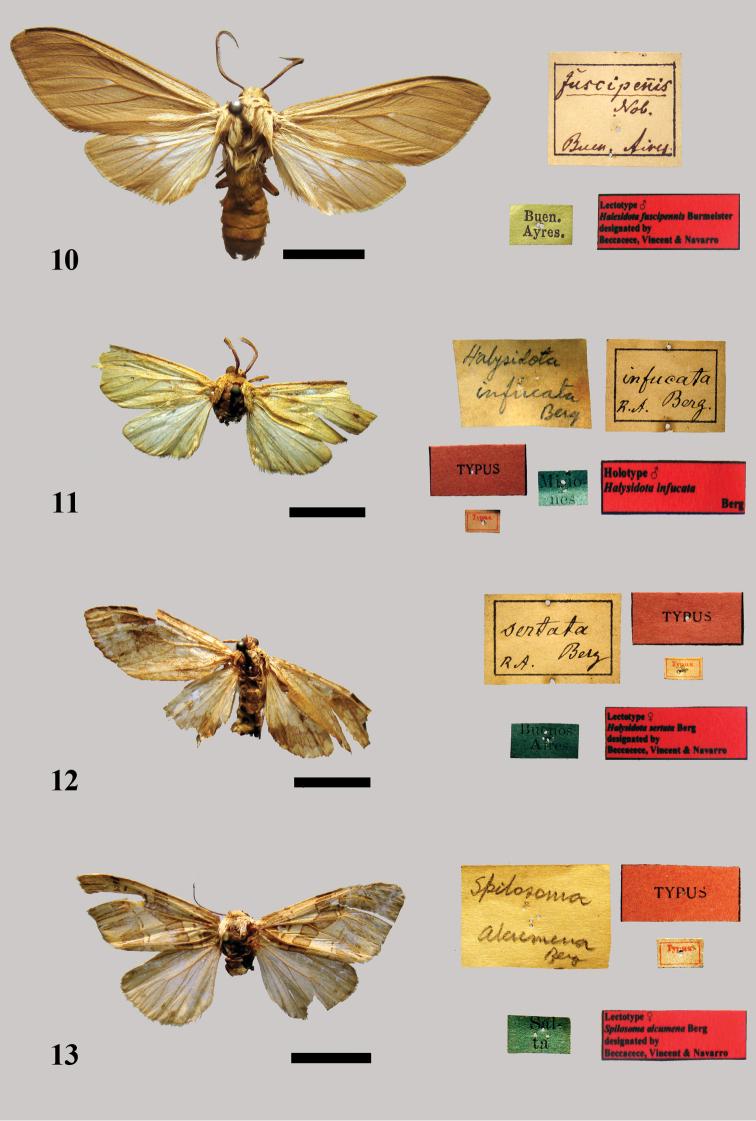
**10** Lectotype male *Halesidota fuscipennis* Burmeister **11** Holotype male *Halysidota infucata* Berg **12** Lectotype female *Halysidota sertata* Berg **13** Lectotype female *Spilosoma alcumena* Berg.

A male syntype included in the Burmeister collection with a green label with the inscription “Buenos Aires” and a red label with the inscription “Lectotype ♂ *Halesidota fuscipennis* Burmeister designated by Beccacece, Vincent & Navarro.” We hereby designate it as lectotype. [MACN]. It is in quite good conditions (Fig. 10). There are two paralectotypes: a male without labels in moderate to good conditions; a male with a green label with the inscription “Buenos Aires,” in moderate to poor condition, with no antennae, the apices of both forewings are missing and the right hindwing is ripped.

##### Remarks.

The lectotype has a similar habitus to *Hypocrisias jonesi* (Schaus, 1894). This taxon was described from an unspecified number of specimens from Castro, Parana (Brazil). A male specimen, housed in USNM, was designated lectotype by Watson (1971). Comparison of the male genitalia of the lectotype dissected by Watson (1971) with the male genitalia from Buenos Aires and the nearby habitus justifies the synonymy of the two taxa. We therefore propose the synonymy given above.

#### Halysidota infucata

Taxon classificationAnimaliaLepidopteraErebidae

Berg, 1882: 216

Fig. 11

##### Current identity.

*Leucanopsis infucata* (Berg, 1882), a junior subjective synonym of *Leucanopsis leucanina* (Felder & Rogenhofer, 1874).

##### Material.

Described from a single male from “Territorios de las Misiones” (Province of Misiones, Argentina).

##### Type locality.

“Territorios de las Misiones” (Province of Misiones, Argentina).

##### MACN.

In the Berg collection there is a male specimen with a blue label “Misiones” / a red label with the inscription “TYPUS,” a white label with the inscription “Typus,” a white label with the inscription“*Halysidota infucata* Berg” (handwritten by Berg). This specimen is therefore the holotype. It is in rather poor conditions with no abdomen and the apices of the forewings are missing (Fig. 11).

##### Remarks.

This species was synonymized with *Leucanopsis leucanina* from Colombia (Felder and Rogenhofer 1874) by Hampson (1901: 166). Based on the analysis of habitus and the type locality of both taxa, we conclude that these species are different and therefore we propose the following recombination: *Leucanopsis infucata* (Berg, 1882), **stat. rev.**

#### Halysidota sertata

Taxon classificationAnimaliaLepidopteraErebidae

Berg, 1882: 214

Fig. 12

##### Current identity.

*Tessella sertata* (Berg, 1882).

##### Material.

Described from one male and one female from “los alrededores [surroundings] de Buenos Aires.”

##### Type locality.

Buenos Aires [Argentina].

##### MACN.

A female syntype in the Berg collection with a green label with the inscription “Buenos Aires.” a red label with the inscription “TYPUS,” a white label with the inscription “Typus” and a red label with the inscription “Lectotype ♀ *Halysidota sertata* Berg designated by Beccacece, Vincent & Navarro.” We hereby designate it as lectotype. [MACN]. It is in very poor condition, with no antennae, both wings are damaged and the right wings are folded backwards (Fig. 12).

##### Remarks.

Berg stated that the male specimen is preserved in the collection of M. Ruscheweyh. In Horn et al. (1990), a part of this collection may be in ZMHB. After verification by the second author, no corresponding specimen was located in this institution.

### Arctiinae, Arctiini, Arctiina

#### Spilosoma alcumena

Taxon classificationAnimaliaLepidopteraErebidae

Berg, 1882: 213

Fig. 13

##### Current identity.

*Isia alcumena alcumena* (Berg, 1882).

##### Material.

Described on the basis of a male specimen from Tucumán (City of San Miguel de Tucuman, Argentina) and a female specimen from Salta (City or province of Salta, Argentina).

##### Type locality.

Salta [Argentina].

##### MACN.

A female syntype housed in the Berg collection with a blue label with the inscription “Salta,” a red label with the inscription “TYPUS,” a white label with the inscription “Typus,” a white label that reads “*Spilosoma alcumena* Berg” (handwritten by Berg), and red label with the inscription “Lectotype ♀ *Spilosoma alcumena* Berg designated by Beccacece, Vincent & Navarro.” It is in bad condition, but it is recognizable: the abdomen and right antenna are missing and both forewing apices and the right hindwing are damaged (Fig. 13). The male, here designated as a paralectotype, bears a blue label with the inscription “Tucuman,” a white label with the inscription “Typus,” a white label with the number “11948” and a white label that reads “*Spilosoma Alcumena* Bov Berg” (handwritten by Berg). It is in moderate to good condition: it is missing the right antenna, the left antenna is broken and left hindwing is ripped.

##### Remarks.

Berg indicated that the male syntype was preserved in Gunther’s collection and the female syntype was in “la Universidad” [MACN], donated by himself. The male paralectotype was also deposited in the MACN.

#### Ecpantheria kinkelini

Taxon classificationAnimaliaLepidopteraErebidae

Burmeister, 1879: 59

Fig. 14

##### Current identity.

*Hypercompe kinkelini* (Burmeister, 1879).

##### Material.

Described on the basis of an unspecified number of male and female specimens from Palermo, near Buénos-Ayres [Buenos Aires].

##### Type locality.

Palermo, Buénos-Ayres [Buenos Aires, Argentina].

##### MACN.

**Figure 14–17.**
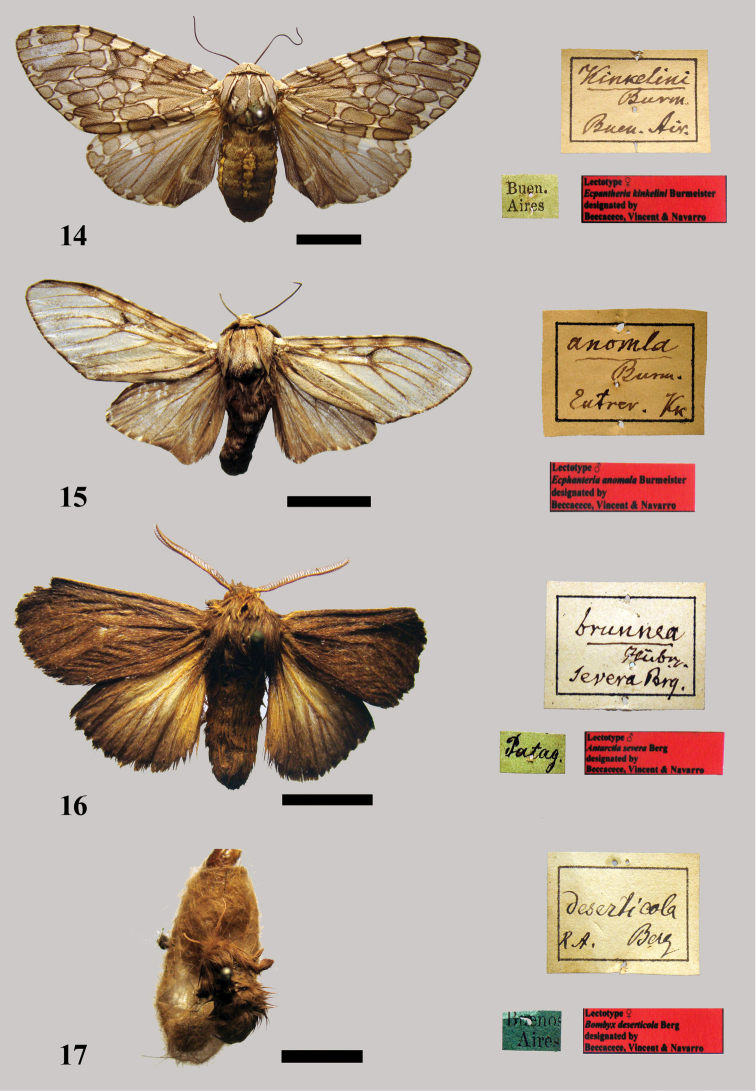
**14** Lectotype female *Ecpantheria kinkelini* Burmeister **15** Lectotype male *Ecpantheria anomala* Burmeister **16** Lectotype male *Antarctia severa* Berg **17** Lectotype female *Bombyx deserticola* Berg.

In the Burmeister collection there are two specimens that apparently belong to the type-series: a male specimen with no label pinned on it; it is in good condition; a female specimen with a green label with the inscription “Buen[os] Aires,” which is in quite good condition (Fig. 14). The habitus and scale of the two specimens correspond to the original description and they are associated with a square label on the box that reads “Kinkelini Burm[eister] Buen[os] Air[es].” Therefore, we assume they are syntypes. We hereby designate the female specimen as lectotype in [MACN] with a red label with the inscription “Lectotype ♀ *Ecpantheria kinkelini* Burmeister designated by Beccacece, Vincent & Navarro.” The male specimen is designated as a paralectotype.

##### Remarks.

The specific name is in honor of Kinkelin (collector of both specimens).

#### Ecpantheria anomala

Taxon classificationAnimaliaLepidopteraErebidae

Burmeister, 1883: 40

Fig. 15

##### Current identity.

*Hypercompe anomala* (Burmeister, 1883).

##### Material.

Described on the basis of an unspecified number of male specimens from Entre Rios [province] (Argentina).

##### Type locality.

Entre Rios [province] [Argentina].

##### MACN.

In the Burmeister collection there is a male specimen with no label pinned on it, but it is associated with a square label in the box that reads “anom[a]la / Burm[eister] / Entrer [Entre Ríos] / Kk [Kinkelin]” (handwritten by Burmeister) and a red label with the inscription “Lectotype ♂ *Ecpantheria anomala* Burmeister designated by Beccacece, Vincent & Navarro.” It presents the habitus and size that match Burmeister's description that feature in the original description. Therefore, we assume that it is a syntype. We hereby designated it as lectotype [MACN]. It is in good condition, with only the right antenna broken (Fig. 15).

#### Antarctia severa

Taxon classificationAnimaliaLepidopteraErebidae

Berg, 1875a: 209

Fig. 16

##### Current identity.

*Paracles severa* (Berg, 1875a).

##### Material.

Described from an unspecified number of specimens from Patagonia (Argentina).

##### Type locality.

Rio Santa Cruz [Santa Cruz province, Argentina]. See Remarks.

##### MACN.

A male syntype included in the Burmeister collection with a green label with the inscription “Patag[onia]” and a red label with the inscription “Lectotype ♂ *Antarctia severa* Berg designated by Beccacece, Vincent & Navarro.” We hereby designate it as lectotype [MACN]. It is in quite good condition, although both tips of the antennae are broken (Fig. 16). We designate one paralectotype, a female specimen with a green label with the inscription “Patag[onia].” It is in moderate to good condition with no antennae, a torn inner base of the left hindwing and a small piece missing from the termen of the right hindwing.

##### Remarks.

Burmeister (1878) indicated the collecting locality of the type specimens more precisely: “Berg discovered this species from Rio Santa Cruz in South Patagonia.”

#### Bombyx deserticola

Taxon classificationAnimaliaLepidopteraErebidae

Berg, 1875a: 212

Fig. 17

##### Current identity.

*Paracles deserticola* (Berg, 1875a).

##### Material.

Described from an unspecified number of female (wingless) specimens from the outfall of the Rio Negro (south of Buenos Aires province).

##### Type locality.

outfall of the Rio Negro [south of Buenos Aires province, Argentina].

##### MACN.

A wingless female syntype included in the Berg collection with a blue label with the inscription “Buenos Aires” and a red label with the inscription “Lectotype ♀ *Bombyx deserticola* Berg designated by Beccacece, Vincent & Navarro.” We hereby designate it as lectotype [MACN]. It is in very poor condition, missing the apical part of the left antenna and a pinned cocoon (Fig. 17). There are two possible paralectotypes: a wingless female with a data label reading “Buenos Aires;” it is almost totally damaged and externally unrecognizable; a wingless female with a data label reading “Buenos Aires,” “Cum typo/Comparat,” which is in moderate to good condition with no antennae. None of these specimens is labelled as a type, which is usual for Berg’s type material. In addition, in the Burmeister collection there are three female specimens: a wingless female with a data label reading “Córdova” [Córdoba], which is in quite good conditions, but the location does not match the location of the type locality; a wingless female with a data label reading “Buen. Ayres.” [*Buenos aires*], which is in quite good conditions, with short antennae; the labelling is by Burmeister; a wingless female with no data label and another label reading “168,” which is in quite good condition and its habitus matches the species description. It is possibly one of the syntypes.

##### Remarks.

Berg (1875a) described *Bombyx deserticola* in German, but without all the information; in a Spanish publication “Lepidópteros Patagonicos observados en el viage de 1874” (The Patagonian Lepidoptera observed in the voyage of 1874) Berg (1875b) indicated that only a female collected from Patagonia and another unspecified number from Buenos Aires were described as *Bombyx deserticola*. Moreover, in the Spanish publication there is a note in the index in which Berg proposes this species as in genus *Trichosoma* Ramb[ur]. (*Ocnogyna* Led[erer].). In addition, in the Berg collection there are three male specimens: a male with labels reading “Typus,” “TYPUS,” “Buenos Aires,” “*Laora deserticola* Berg” (handwritten by Berg). It is in moderate condition with the distal half of the right antenna broken; a male specimen with labels reading “Cumtypo/comparat.” and “Buenos Aires,” which is in good condition; a male labelled “Cumtypo/comparat.,” “Buenos Aires,” which is in poor condition with no abdomen and the distal of the right antenna broken. These three male specimens cannot be paralectotypes because the description is based only on wingless females.

#### Palustra azollae

Taxon classificationAnimaliaLepidopteraErebidae

Berg, 1877a: 258

Fig. 18

##### Current identity.

*Paracles azollae* (Berg, 1877).

##### Material.

Described from a male specimen from Tigre (north of Buenos Aires), 15 specimens from the south of Buenos Aires, and an unspecified number of specimens from the Riachuelo channels, close to the mouth of the Rio de la Plata. All the specimens were reared from caterpillars.

##### Type locality.

Buenos Aires [Argentina].

##### MACN.

**Figure 18–21.**
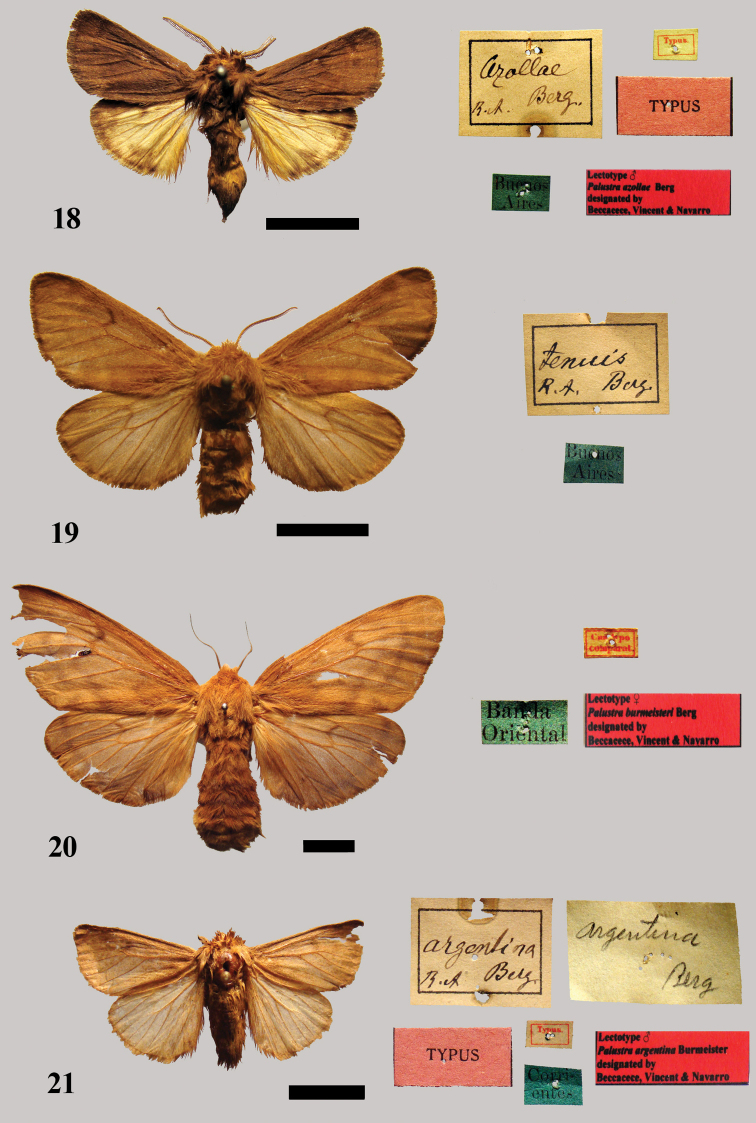
**18** Lectotype male *Palustra azollae* Berg **19** Possible syntype female of *Palustra tenuis* **20** Lectotype female *Palustra burmeisteri* Berg **21** Lectotype male *Palustra argentina* Berg.

a male syntype included in the Berg collection with a blue label with the inscription “Buenos Aires,” a red label with the inscription “TYPUS,” a white label with the inscription “Typus” and a red label with the inscription “Lectotype ♂ *Palustra azollae* Berg designated by Beccacece, Vincent & Navarro.” We hereby designate it as lectotype [MACN]. It is in moderate condition, missing the apical half of the right antenna (Fig. 18). We designate two paralectotypes: (1) a male with red label with the inscription “TYPUS,” a blue label with the inscription “Buenos Aires” and a white label “*azollae* Berg” (handwritten by Berg), which is in quite good condition; (2) a female specimen with a blue label with the inscription “Buenos Aires,” a red label with the inscription “TYPUS” and a white label with the inscription “Typus.” It is in moderate to poor condition, missing the right forewing and the right hindwing is damaged.

##### Remarks.

In the Burmeister collection there are six specimens: a male specimen with a label “Buen. Ayres” [Buenos Aires], which is in quite good condition; a male without labels, which is in quite good condition, although the right antenna is missing; a male without labels, which is in moderate condition: the antennae are missing; a female with a label “Buen. Ayres” [Buenos Aires], which is in moderate condition because the abdomen is missing; a female without labels in good condition; a female without labels in good condition. These labelled specimens are not syntypes because they are labelled in Burmeister’s manner. On the other hand, we cannot confirm whether or not the other unlabelled specimens are types.

*Paracles azollae* Berg, 1877a was redescribed under the same name as new by Berg (1877b: 191).

#### Palustra tenuis

Taxon classificationAnimaliaLepidopteraErebidae

Berg, 1877a: 259

Fig. 19

##### Current identity.

*Paracles tenuis tenuis* (Berg, 1877).

##### Material.

Described from a male and two females from Boca de Riachuelo [outfall of Riachuelo] near Buenos Ayres [Buenos Aires].

##### Type locality.

outfall of Riachuelo, near Buenos Ayres [Buenos Aires, Argentina].

##### MACN.

In the Berg collection there is a female specimen with a data label reading “Buenos Aires;” it is in quite good condition, although the right forewing is ripped (Fig. 19). This specimen is not labelled as type, as Berg’s type material usually is, however, it could be one of the female syntypes.

##### Remarks.

In the Burmeister collection there are three specimens labelled as *Palustra tenuis* Berg: a male specimen with a label “Buen. Ayres” [Buenos Aires], which is in quite good condition, although it has no antennae; a female specimen with no data label, a small piece missing at the right apex of the forewing as well as the left antenna; a female specimen with the data label “Buen. Ayres” [Buenos Aires], which is in quite good conditions, although a small piece is missing at the right apex of the forewing and the left hindwing is ripped. These specimens are not likely to be syntypes because they are not labelled in Berg’s manner. The aged condition and the decolored pattern of the possible female lectotype in the Berg collection could be confused with the female of *Paracles azollae* (Berg).

*Palustra tenuis* Berg, 1877a was redescribed under the same name as new by Berg (1877b: 191).

#### Palustra burmeisteri

Taxon classificationAnimaliaLepidopteraErebidae

Berg, 1877c: 228

Fig. 20

##### Current identity.

*Paracles burmeisteri* (Berg, 1877).

##### Material.

Described from a female and a male (atrophied) specimen obtained after rearing caterpillars from the “Banda Oriental” [Eastern Band of Uruguay].

##### Type locality.

Banda Oriental [Eastern Band, Republic of Uruguay].

##### MACN.

In the Berg collection there are two specimens from the type locality: a female specimen with data labels reading “Cumtipo/comparat.,” “Banda Oriental.” It is in moderate to good conditions with the apex of the left forewing torn (Fig. 20). Its habitus is exactly like figure 1 in the original description. We hereby designate it as lectotype [MACN]. This female is labelled with a red label with the inscription “Lectotype ♀ *Palustra burmeisteri* Berg designated by Beccacece, Vincent & Navarro;” a male specimen with data labels reading “Banda oriental,” “*burmeisteri* Berg” (handwritten ), in moderate to good conditions with no antennae nor right wing. This specimen does not bear a label of type. In the Burmeister collection there are two male and two female specimens without labels. There are only two labels in the box: “*burmeisteri* Berg, Rep. Urug [Republic of Uruguay]” and “*Palustra* Don. [donated by] Berg.” One of the male specimens is clearly atrophied. We assume that it is the other syntype (the atrophied male), we hereby designate it as a paralectotype.

##### Remarks.

In the Berg collection the female specimen is in moderate to good condition, although it is missing a considerable portion of the apex of the right forewing and left hindwing, and bears no labels. The other male and two females in the Burmeister collection are not syntypes.

*Palustra burmeisteri* Berg, 1877c was redescribed under the same name as new by Berg (1878: 224).

#### Palustra argentina

Taxon classificationAnimaliaLepidopteraErebidae

Berg, 1877c: 233

Fig. 21

##### Current identity.

*Paracles argentina* (Berg, 1877). It is a junior subjective synonym of *Paracles laboulbeni* (Bar, 1873).

##### Material.

Described on the basis of six specimens from Corrientes (province of Corrientes, Argentina).

##### Type locality.

Corrientes [province of Corrientes, Argentina].

##### MACN.

a male syntype included in the Berg collection with a blue label with the inscription “Corrientes,” a red label with the inscription “TYPUS,” a white label with the inscription “Typus” and a white label with “*argentina* Berg” (handwritten by Berg), and a red label with the inscription “Lectotype ♂ *Palustra argentina* Berg designated by Beccacece, Vincent & Navarro.” We hereby designate it as lectotype [MACN]. It is in moderate to good conditions, although it has no antennae and the apex of the right forewing is torn (Fig. 21). From the Burmeister collection we designate two paralectotypes: a male specimen with a blue label printed “Corrientes” and a white label with the inscription “Typus;” a female specimen with a blue label with the inscription “Corrientes” and a white label with the inscription “Typus.” Both specimens are in moderate to good overall condition, although rather worn.

##### Remarks.

In the Berg collection, there is a female specimen with a blue label with the inscription “Misiones,” a red label with the inscription “TYPUS” and a white label with the inscription “Typus.” It is in bad condition as it is missing the abdomen and the right antenna and the apices of both wings are damaged; according to the locality data, this specimen was incorrectly labelled as a type. *Paracles argentina* (Berg, 1877) was synonymized with *Paracles laboulbeni* (Bar, 1873) by Hampson (1901: 512). This latter species is from French Guiana and is characterized by the forewings having three transverse bands with little contrast. This feature is absent in the lectotype of *Paracles argentina*, so we revise the status of this species: *Paracles argentina* (Berg, 1877), **sp. rev.**

*Palustra argentina* Berg, 1877c was redescribed under the same name as new by Berg (1878: 227).

#### Antarctia persimilis

Taxon classificationAnimaliaLepidopteraErebidae

Burmeister, 1878: 452

Fig. 22

##### Current identity.

*Paracles persimilis* (Burmeister, 1878).

##### Material.

Described on the basis of a male and a female specimen from Rio de Janeiro (Brazil).

##### Type locality.

Rio de Janeiro, Brasil.

##### MACN.

**Figure 22–25.**
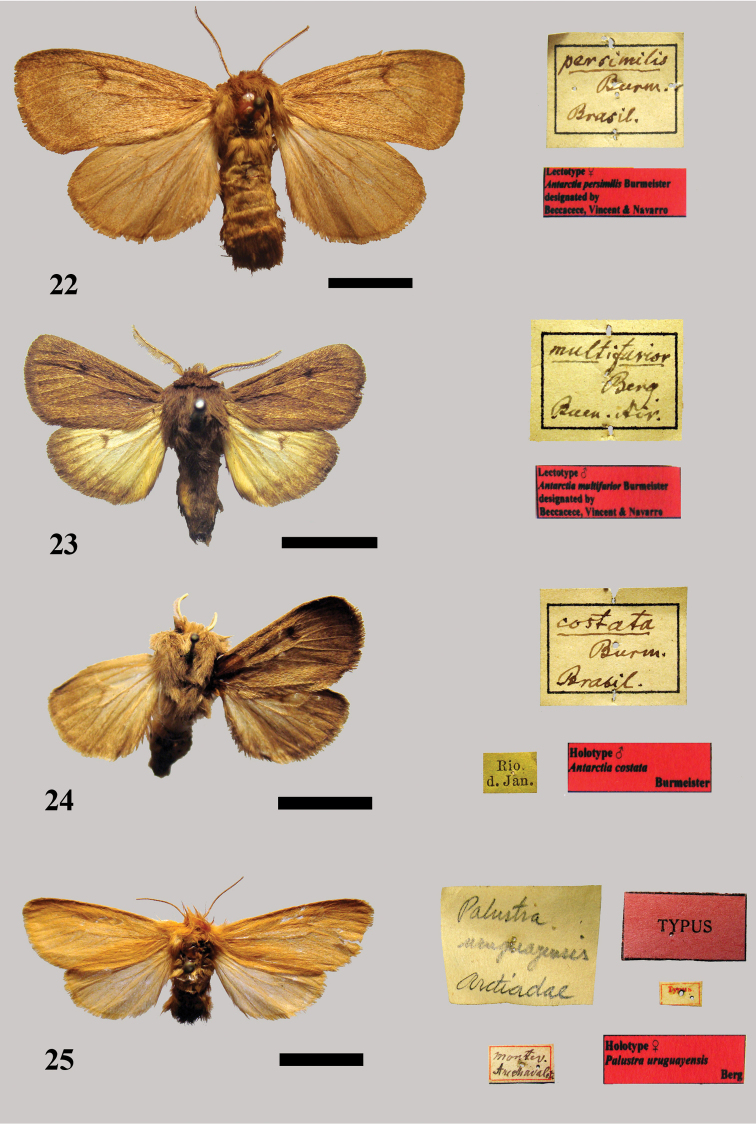
**22** Lectotype female *Antarctia persimilis* Burmeister **23** Lectotype male *Anarctia multifarior* Burmeister **24** Holotype male *Anarctia costata* Burmeister **25** Holotype female *Palustra uruguayensis* Berg.

In the Burmeister collection there is a single female specimen with no label pinned on it (Fig. 22). The only label associated with the specimen is the square white label with “*persimilis* Burm[eister] Brasil.” (Burmeister’s handwriting) and a red label with the inscription “Lectotype ♀ *Antarctia persimilis* Burmeister designated by Beccacece, Vincent & Navarro.” It is inferred that the female specimen belongs to the original type series and we hereby designate it as lectotype [MACN]. It is in good overall condition.

##### Remarks.

The male syntype was not located.

#### Antarctia multifarior

Taxon classificationAnimaliaLepidopteraErebidae

Burmeister, 1878: 452

Fig. 23

##### Current identity.

*Paracles multifarior* (Burmeister, 1878), a junior subjective synonym of *Paracles fusca* (Walker, 1856).

##### Material.

Described on the basis of an unspecified number of specimens from Buénos-Ayres [Buenos Aires].

##### Type locality.

Buénos-Ayres [Buenos Aires, Argentina].

##### MACN.

In the Burmeister collection there are two male specimens with no label pinned on them. They are in quite good condition and have little information on the square label: “*multifarior* Berg Buen[os] Air[es]” (Burmeister’s handwriting) (Fig. 23). We hereby designate the first male specimen as lectotype in [MACN] with a red label with the inscription “Lectotype ♂ *Anctartia multifarior* Burmeister designated by Beccacece, Vincent & Navarro.” The other specimen is designated as a paralectotype.

##### Remarks.

There is a female of *Paracles azollae* (Berg, 1877) incorrectly placed in the series below the main label.

#### Antarctia costata

Taxon classificationAnimaliaLepidopteraErebidae

Burmeister, 1878: 451

Fig. 24

##### Current identity.

*Paracles costata* (Burmeister, 1878), senior synonym of *Paracles bergi* (Schaus, 1896).

##### Material.

Described on the basis of a single specimen from Rio de Janeiro (Brazil).

##### Type locality.

Rio de Janeiro [Brazil].

##### MACN.

In the Burmeister collection there is a male specimen with a green label with the inscription “Rio d[e] Jan[eiro];” it is in moderate to good condition, but missing the left forewing (Fig. 24). Its habitus matches the original description and for this reason we believe that this specimen is the holotype. We label it with a white label with the inscription “Holotype ♂ *Antarctia costata* Burmeister.”

#### Palustra uruguayensis

Taxon classificationAnimaliaLepidopteraErebidae

Berg, 1886: 212

Fig. 25

##### Current identity.

*Paracles uruguayensis* (Berg, 1886) is a junior synonym of *Paracles vulpina* (Hübner, [1825]).

##### Material.

Described on the basis of a single female specimen from Respublica uruguayensis [Republic of Uruguay] Montevideo (Uruguay).

##### Type locality.

Montevideo [Uruguay].

##### MACN.

In the Berg collection there is a female specimen with a white label which reads “Montev[ideo] Arechavaleta col,” a red label with the inscription “TYPUS,” a white label with the inscription “Typus,” a white label that reads “*Palustra uruguayensis* arctiadae” (handwritten by Berg) and a red label with the inscription “Holotype ♀ *Palustra uruguayensis* Berg.” According to the original description, this female is the holotype. We labelled it and herewith recognize it as the holotype [MACN]. It is in good overall condition (Fig. 25).

##### Remarks.

This species was synonymized with *Paracles vulpina* (Hübner, [1825]) by Hampson (1901: 448). Superficially, the figures of *Antarctia vulpina* Hübner and the holotype of *Paracles uruguayensis* Berg have quite different appearances. The head and thorax of the male *Antarctia vulpina* are bright red brown, the axis of the antenna is white; the abdomen has an orange dorsum. The forewing is bright red brown, the costal edge is yellowish white, and the cilia are white at the tips. The hindwings are white, the base of the inner area has some orange hair, the costa and veins towards the apex are brownish. The forewing of the female has a slightly paler costa, and the cilia are not white at the tips. The hindwing is pale brown (Hampson, 1901). The holotype female of *Paracles uruguayensis* Berg has a pale grey-yellow color pattern. The species is similar to *Paracles laboulbeni* (Bar, 1873). Therefore, we propose the two taxa are valid species with the following recombination: *Paracles uruguayensis* (Berg, 1886), **sp. rev.**

### Arctiinae, Arctiini, Pericopina

#### Eucharia centenaria

Taxon classificationAnimaliaLepidopteraErebidae

Burmeister, 1878: 436

Fig. 26

##### Current identity.

*Dysschema centenaria centenaria* (Burmeister, 1878).

##### Material.

Described from a single male from Zárate (Province of Buenos Aires, Argentina).

##### Type locality.

Zárate [Province of Buenos Aires, Argentina].

##### MACN.

In the Berg collection there is a male specimen with the following labels: “Cumtypo/comparat.” and “Zarate Kink[elin].” It could be the male holotype; it is in quite good overall condition (Fig. 13).

**Figure 26.**
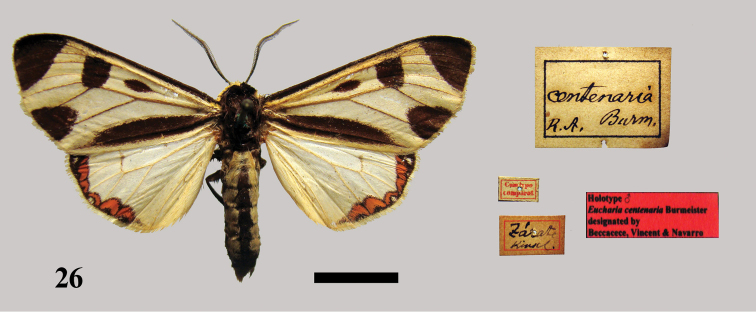
Holotype male *Eucharia centenaria* Burmeister.

##### Remarks.

In the original description Burmeister stated that the taxon was discovered and named by M. Kinkelin. We have not found a publication of Kinkelin describing the taxon. Burmeister apparently was the first to name and describe this taxon and is the sole author. The male specimen labelled “Cumtypo/comparat.” is probably the male holotype. The original description, type locality and collector are identical for this specimen.

## Supplementary Material

XML Treatment for Halesidota picturata

XML Treatment for Halesidota cancellata

XML Treatment for Halesidota rectilinea

XML Treatment for Halesidota trifasciata

XML Treatment for Halesidota fuscipennis

XML Treatment for Halysidota infucata

XML Treatment for Halysidota sertata

XML Treatment for Spilosoma alcumena

XML Treatment for Ecpantheria kinkelini

XML Treatment for Ecpantheria anomala

XML Treatment for Antarctia severa

XML Treatment for Bombyx deserticola

XML Treatment for Palustra azollae

XML Treatment for Palustra tenuis

XML Treatment for Palustra burmeisteri

XML Treatment for Palustra argentina

XML Treatment for Antarctia persimilis

XML Treatment for Antarctia multifarior

XML Treatment for Antarctia costata

XML Treatment for Palustra uruguayensis

XML Treatment for Eucharia centenaria
